# Motor, cognitive and behavioural profiles of *C9orf72* expansion-related amyotrophic lateral sclerosis

**DOI:** 10.1007/s00415-022-11433-z

**Published:** 2022-10-29

**Authors:** Eleonora Colombo, Barbara Poletti, Alessio Maranzano, Silvia Peverelli, Federica Solca, Claudia Colombrita, Silvia Torre, Cinzia Tiloca, Federico Verde, Ruggero Bonetti, Laura Carelli, Claudia Morelli, Antonia Ratti, Vincenzo Silani, Nicola Ticozzi

**Affiliations:** 1 Department of Neurology and Laboratory of Neuroscience, IRCCS Istituto Auxologico Italiano, Università degli Studi di Milano, P.le Brescia 20, 20149 Milan, Italy; 2 “Dino Ferrari Center”, Department of Pathophysiology and Transplantation, Università degli Studi di Milano, 20122 Milan, Italy; 3 Department of Medical Biotechnology and Translational Medicine, Università degli Studi di Milano, 20122 Milan, Italy

**Keywords:** ALS, Frontotemporal dementia, Genetics, Motor neuron disease

## Abstract

**Introduction:**

Amyotrophic lateral sclerosis (ALS) individuals carrying the hexanucleotide repeat expansion (HRE) in the *C9orf72* gene (C9Pos) have been described as presenting distinct features compared to the general ALS population (C9Neg). We aim to identify the phenotypic traits more closely associated with the HRE and analyse the role of the repeat length as a modifier factor.

**Methods:**

We studied a cohort of 960 ALS patients (101 familial and 859 sporadic cases). Motor phenotype was determined using the MRC scale, the lower motor neuron score (LMNS) and the Penn upper motor neuron score (PUMNS). Neuropsychological profile was studied using the Italian version of the Edinburgh Cognitive and Behavioral ALS Screen (ECAS), the Frontal Behavioral Inventory (FBI), the Beck Depression Inventory-II (BDI-II) and the State-Trait Anxiety Inventory (STAI). A two-step PCR protocol and Southern blotting were performed to determine the presence and the size of *C9orf72* HRE, respectively.

**Results:**

*C9orf72* HRE was detected in 55/960 ALS patients. C9Pos patients showed a younger onset, higher odds of bulbar onset, increased burden of UMN signs, reduced survival and higher frequency of concurrent dementia. We found an inverse correlation between the HRE length and the performance at ECAS ALS-specific tasks (*P* = 0.031). Patients also showed higher burden of behavioural disinhibition (*P* = 1.6 × 10^–4^), lower degrees of depression (*P* = 0.015) and anxiety (*P* = 0.008) compared to C9Neg cases.

**Conclusions:**

Our study provides an extensive characterization of motor, cognitive and behavioural features of *C9orf72*-related ALS, indicating that the *C9orf72* HRE size may represent a modifier of the cognitive phenotype.

**Supplementary Information:**

The online version contains supplementary material available at 10.1007/s00415-022-11433-z.

## Introduction

Amyotrophic lateral sclerosis (ALS) is a fatal neurodegenerative disorder characterized by a progressive loss of upper (UMN) and lower motor neurons (LMN). Approximately 90% of ALS cases are sporadic (SALS), while the remaining 10% are familial (FALS). Mutations in four main genes (*C9orf72*, *SOD1*, *TARDBP* and *FUS*) are responsible for up to 75% of FALS cases, with variants in > 25 other genes being relatively uncommon [1].

A (G_4_C_2_)_n_ hexanucleotide repeat expansion (HRE) in the *C9orf72* gene accounts for 30–50% of FALS, as well as 5–10% of SALS cases [2], and represents the most common genetic defect in ALS and in frontotemporal dementia (FTD) [3, 4].

ALS individuals carrying the *C9orf72* HRE (C9Pos) often present different phenotypic traits compared to the remaining patients (C9Neg), showing a more aggressive form of the disease characterized by a higher prevalence of bulbar onset, earlier age at onset, and reduced survival [2, 4–8]. C9Pos cases also have an increased frequency of comorbid frontotemporal dementia (ALS-FTD) and a higher prevalence of a family history of ALS and/or other neurodegenerative diseases [2, 5, 8, 9]. Intriguingly, the same HRE length may cause ALS, FTD or mixed ALS-FTD manifestations with no clear genotype–phenotype correlations [10, 11]. However, the current molecular diagnosis of *C9orf72* HRE by repeat-primed PCR (RP-PCR) gives no information on HRE length above a certain threshold (50–150 repeats), and the difficulty in performing and including Southern blot assay in a clinical setting has made accurate genotype–phenotype correlations inconclusive. Moreover, in contrast to other repeat expansion disorders, C9Pos patients show neither consistent association between HRE size and disease severity nor clear genetic anticipation [12].

The recognition of C9Pos individuals is a key issue, both for stratification in randomized clinical trials and because the development of antisense oligonucleotide-based therapies is already underway [13]. In fact, the presence of *C9orf72* HRE is an independent predictor of shorter survival in ALS, and it has been included in prediction models [14]. Here we studied a large cohort of Italian ALS patients with the aim of identifying the phenotypic traits more closely associated with *C9orf72* HRE and to assess any possible correlations of the HRE length with motor, cognitive and behavioural features.

## Patients and methods

### Patients

A cohort of 960 Italian patients diagnosed with ALS and other motor neuron diseases (primary lateral sclerosis [PLS] and progressive muscular atrophy [PMA]) according to El Escorial revised criteria [15] was consecutively recruited at a tertiary ALS Centre (IRCCS Istituto Auxologico Italiano, Milan, Italy) between 2008 and 2021. The following data were collected: sex; age at onset; family history of ALS, dementia and/or parkinsonism; site of onset; phenotype; ALSFRS-R score at evaluation; progression rate; clinical stages according to the King and Milano-Torino (MITOS) staging systems [16]; survival.

### Standard protocol approvals and patients’ consent

Informed consent for using pseudo-anonymized data for research purposes was obtained from all patients or their authorized legal representatives. This study was approved by the Ethics Committee of Istituto Auxologico Italiano IRCCS (project DAMARE 2021_05_18) and conducted according to the principles expressed in the Declaration of Helsinki.

### Evaluation of motor phenotype

The burden of UMN and LMN involvement was determined using three different semi-quantitative scales, namely the MRC (Medical Research Council) scale for muscle strength, the LMN score (LMNS) [17], and the Penn UMN score (PUMNS) [18]. For MRC the strength of three different muscle groups for each limb (shoulder abductors, elbow flexors, wrist dorsiflexors, hip flexors, knee extensors and ankle dorsiflexors) was assessed. The LMNS defines the burden of LMN signs in each limb (0–3), with higher scores corresponding to greater impairment [17]. The score was modified to consider the presence of LMN signs also in the thoracic and bulbar regions, assigning 1 point each, for a total value ranging from 0 to 14. Lastly, PUMNS was used to define the severity of UMN impairment in the bulbar region (score 0–4) and each limb (score 0–7), for a total score of 0–32.

### Neuropsychological testing

The cognitive profile was investigated using the Italian version of the Edinburgh Cognitive and Behavioural ALS Screen (ECAS) [19] and the Frontal Behavioural Inventory (FBI) [20]. Comorbid FTD was determined according to the criteria proposed by Neary [21] and Rascovsky [22]. Patients were subsequently classified according to the Strong revised criteria [unimpaired, behavioural (ALSbi), cognitive (ALSci), cognitive and behavioural impairment (ALScbi), ALSFTD] [23]. For cognitive status assessment, the following scores were considered: ECAS total, ALS-Specific, ALS-Nonspecific, as well as the single ECAS subdomains (executive, verbal fluency, language, memory, visuospatial) scores. The behavioural profile was evaluated considering the symptoms detected at the ECAS Carer Interview (disinhibition, apathy/inertia, loss of sympathy/empathy, perseverative/stereotyped/compulsive/ritualistic behaviour and hyperorality/altered food preferences), as well as the scores at FBI-A (negative behaviours) and FBI-B (positive/disinhibited behaviours). Psychological status was explored with the Beck Depression Inventory-II (BDI-II; cognitive-affective and somatic symptoms) [24] and the State-Trait Anxiety Inventory (STAI; Y1 state anxiety, Y2 trait anxiety) [25]

### *C9orf72* genetic analysis

Genetic analysis of *C9orf72* was performed on DNA extracted from peripheral blood using a two-step PCR protocol, including a first fluorescent amplicon-length analysis with primers designed in the unique sequence flanking the hexanucleotide repeat (FAM-labelled forward 5′-TGTAAAACGACGGCCAGTCAAGGAGGGAAACAACCGCAGCC-3′ and reverse 5′-GCAGGCACCGCAACCGCAG-3′) and, only for samples showing one fluorescent peak, the Repeat-PCR (RP-PCR) was carried out, as previously described [10]. For both amplicon length analysis and RP-PCR, amplicons were run on ABI Prism 3500 Genetic Analyzer (Applied Biosystems) and visualized using Gene Mapper v.4 software (Applied Biosystems). A cut-off value of > 30 repeats was used to define the pathogenic threshold in RP-PCR assay.

### Southern blotting

Genomic DNA (12 μg) was digested with XbaI restriction enzyme and run on 0.7% agarose gel. Southern blot was performed using a unique sequence probe, mapping within *C9orf72* first intron and obtained by PCR amplification with the following primers: forward 5′-CTTTCTCCAGATCCAGCAGCCTCC-3′ and reverse 5′-CTGAGTTCCAGAGCTTGCTACAG-3′. Upon probe labelling with dCTP^32^, hybridized membranes were resolved by autoradiography as previously described [10]. For each sample, the HRE size was calculated as mode of the observed smear range [26].

### Statistical analysis

Descriptive statistics are reported as numbers and percentages for categorical variables or mean, median and standard deviation for continuous variables. Statistical analysis was carried out with IBM Statistical Package for Social Science (SPSS) version 26. Univariate survival analysis was performed with Kaplan–Meier curves followed by log-rank test. Multivariate survival analysis was performed with Cox regression, using *C9orf72* status, age at onset, site of onset and gender as covariates. Differences in *C9orf72* status for categorical variables were assessed with Pearson’s *χ*^2^ test or Fisher exact test when appropriate. Mann–Whitney *U* test was used for continuous variables. Pearson bivariate correlation was performed to evaluate the relationship between HRE size and continuous variables. Two-tailed *P* values < 0.05 were considered significant. Listwise deletion was used to exclude cases missing *C9orf72* HRE status. Pairwise deletion was used to handle missing data for correlation with other phenotypic traits.

### Data availability

Pseudo-anonymized data are archived on Zenodo and will be disclosed upon reasonable request (doi:10.5281/zenodo.6245606).

## Results

### Clinical and genetic characteristics of the ALS cohort

We recruited a cohort of 960 Italian ALS patients, 37.2% (357) of whom were females, and 62.8% (603) males. The mean age at onset was 59.3 years (± 12.3) while median survival was 48.4 months (± 38.9). Site of onset was bulbar in 231 (24.1%) and spinal in 729 (75.9%) patients. Clinical phenotype could be determined in 955 cases, 495 (51.8%) of which had classic, 204 (21.4%) bulbar and 19 (2.0%) respiratory ALS. LMN-predominant phenotypes were observed in 42 (4.4%) patients with PMA, 42 (4.4%) with flail arm and 24 (2.5%) with flail leg syndrome. Conversely, 89 (9.3%) patients had UMN-predominant ALS and 40 (4.2%) had PLS. FALS cases accounted for 10.5% (*n* = 101) of our cohort. A positive family history for dementia or Parkinsonism was observed in 14.8% and 7.2% cases, respectively.

**Fig. 1:**
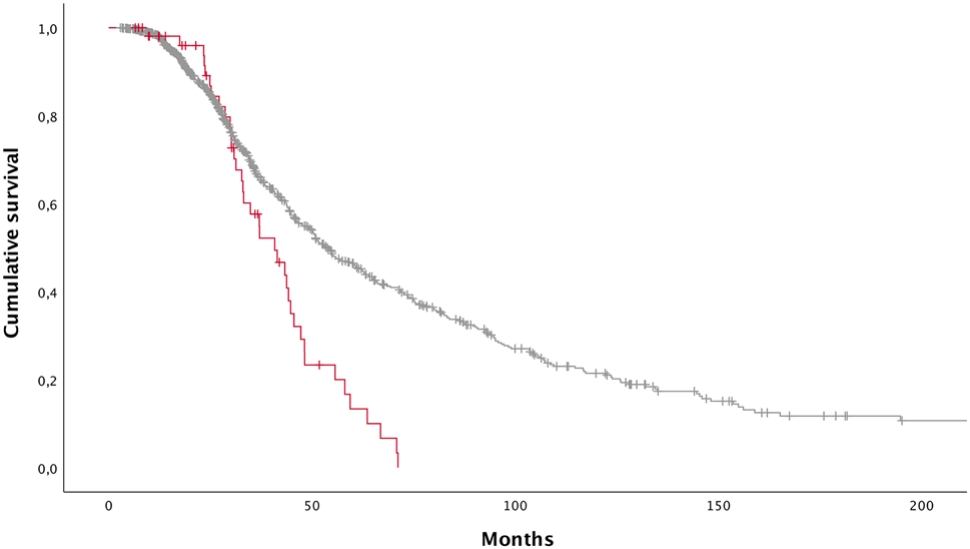
Kaplan–Meier plots of survival probabilities. Patients carrying the pathogenic *C9orf72* repeat expansion (C9Pos, red line) display shorter survival time compared to patients without the genetic mutations (C9Neg, grey line); + : censored cases

By a two-step protocol with fragment length analysis and Repeat-primed PCR, we identified the presence of the *C9orf72* HRE in 55 (5.7%) patients, of whom 19 were FALS (18.8%) and 36 SALS (4.2%), in line with reported mutational frequencies in the Italian population [4]. C9Pos patients showed an earlier age at onset (53.5 vs 61.2 years, *P* = 3.5 × 10^–4^) and a reduced survival compared to C9Neg individuals at univariate [40.8 vs 53.5 months, *P* = 7.1 × 10^–5^, hazard ratio [HR] 2.0 (95% confidence interval [CI] 1.4–2.8)] (Fig. 1) and multivariate analysis [*P* = 2.0 × 10^–6^, HR 2.3 (95% CI = 1.6–3.3)] (Supplementary Table 1). Mutated patients also displayed increased frequency of bulbar onset [21/55 vs 210/905; 38.1% vs 23.2%; odds ratio [OR] 2.0 (95% CI 1.2–3.6); *P* = 0.012] and more often a bulbar ALS compared to other phenotypes [20/55 vs 184/900; 36.3% vs 20.4%; OR 2.2 (95% CI = 1.3–3.9); *P* = 0.005] (Table 1). Moreover, C9Pos patients more likely had a positive family history for dementia [18/48 vs 93/702; 37.5% vs 13.2%; OR 3.9 (95% CI 2.1–7.3); *P* = 5.0 × 10^–6^], but not for Parkinsonism (4/48 vs 50/702; 8.3% vs 7.1%; *P* > 0.05). We did not observe any difference between C9Pos and C9Neg individuals regarding sex distribution, functional impairment as measured by the ALSFRS-R, time to placement of NIV and/or PEG, disease progression and staging (Table 1).

**Table 1:** Demographic and clinical characteristics of the study cohorts: comparison between C9Pos and C9Neg patients

	C9Pos				C9Neg				*P* value^a^	Total
	*n*	%	Mean	Median	*n*	%	Mean	Median		
Site of onset										
Bulbar	21	38.2			210	23.2			0.012	231
Spinal	34	61.8			695	76.8				729
Phenotype										
Classic	25	45.5			470	51.9			ns	495
Bulbar	20	36.4			184	20.3			0.005	204
Respiratory	0	0.0			19	2.1			ns	19
Flail arm	1	1.8			41	4.5			ns	42
Flail leg	1	1.8			23	2.5			ns	24
PMA	0	0.0			42	4.6			ns	42
UMN predominant	7	12.7			82	9.0			ns	89
PLS	1	1.8			39	4.3			ns	40
ALS family history										
FALS	19	34.5			82	9.1			2.2 × 10^−9^	101
SALS	36	65.5			823	90.9				859
Dementia family history										
Yes	18	37.5			93	13.2			5.0 × 10^−6^	111
No	30	62.5			609	86.8				639
Parkinsonism family history										
Yes	4	8.3			50	7.1			ns	54
No	44	91.7			652	92.9				696
King staging										
1–2	9	19.1			209	26.9			ns	218
3–4	38	80.9			567	73.1				605
Age at onset			54.9	53.5			59.7	61.2	3.5 × 10^−4^	
Time to DNA collection			18.3	16.4			20.0	14.7	ns	
Time to NIV			33.7	31.2			33.5	24.5	ns	
Time to PEG			45.1	39.3			33.5	24.5	ns	
ALSFRS-R			35.8	39.0			37.1	39.0	ns	
Progression rate			0.86	0.69			0.84	0.60	ns	
Survival			39.3	40.8			49.3	53.5	7.1 × 10^−5^	

ns, not significant; C9Pos, patients carrying *C9orf72* repeat expansion; C9Neg, patients without *C9orf72* repeat expansion; PEG, percutaneous endoscopic gastrostomy; NIV, noninvasive ventilation; UMN, upper motor neuron; PLS, primary lateral sclerosis; PMA, progressive muscular atrophy; ALS, amyotrophic lateral sclerosis; FALS, familial ALS; SALS, sporadic ALS. a: comparison between C9Pos and C9Neg patients

**Fig. 2:**
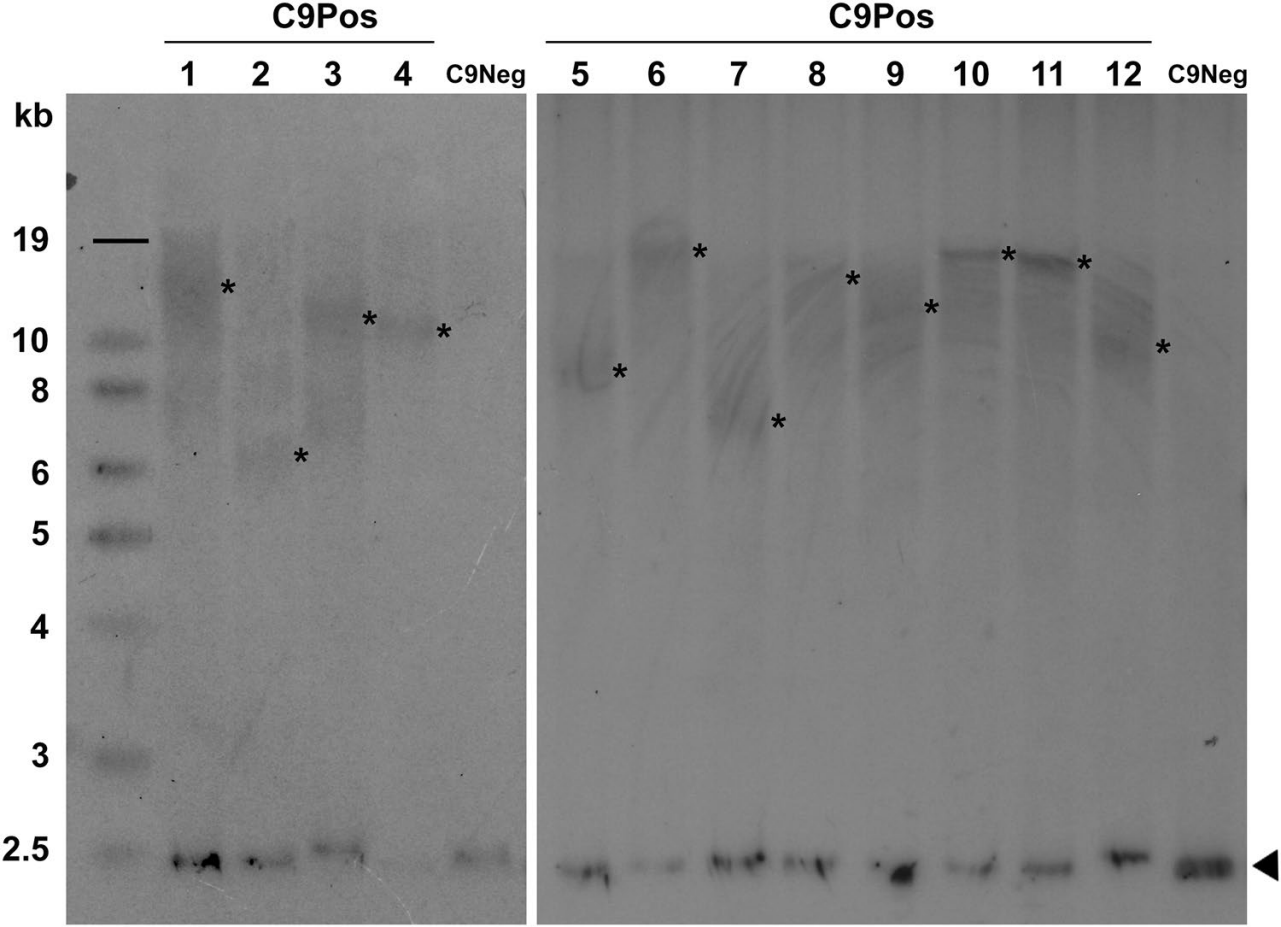
Representative Southern blot images of 12 different C9Pos blood DNA samples. A C9Neg sample was also included as negative control. The mode value within the smear range in each lane was considered for HRE size (Asterisks). Arrowheads indicate the *C9orf72* wild-type allele (2.3 Kb). C9Pos, patients carrying *C9orf72* repeat expansion; C9Neg, patients without *C9orf72* repeat expansion

To better assess genotype–phenotype correlations in ALS patients carrying the *C9orf72* gene mutation, we determined HRE size by Southern blot in 46/55 C9Pos blood samples. All the C9Pos samples identified by RP-PCR were confirmed as carrying a HRE by Southern blot assay. Our analysis revealed high HRE mosaicism, with G_4_C_2_ repeats ranging from about 600 to 7000 units (average length ~ 2150 units) (Fig. 2). When we correlated HRE size with age at onset, survival and other clinical parameters, no significant associations were identified (data not shown).

### Evaluation of the motor phenotype

To assess the contribution of the *C9orf72* HRE to the burden of UMN and LMN signs, we used three different clinimetric scales. PUMNS, LMNS and MRC scores were available for 822 [46/55 (83.7%) C9Pos, 776/905 (85.7%) C9Neg], 821 [46/55 (83.7%) C9Pos, 775/905 (85.6%) C9Neg] and 631 [37/55 (67.3%) C9Pos, 594/905 (65.6%) C9Neg] patients respectively. C9Pos patients displayed a motor phenotype characterized by more prominent UMN signs, as measured by the PUMNS, compared to C9Neg cases (12.9 ± 7.6 vs 10.0 ± 7.3; *P* = 0.010) (Supplementary Fig. 1a). PUMNS scores were higher both for the bulbar (1.9 ± 1.3 vs 1.4 ± 1.2; *P* = 0.005) and spinal regions (11.1 ± 6.9 vs 8.6 ± 6.8; *P* = 0.016) (Table 2, Supplementary Fig. 1b, c). Among bulbar signs the strongest association with the HRE was found for jaw reflex [27/46 vs 257/776; 58.7% vs 33.1%; OR 2.9 (95% CI 1.6–5.3); *P* = 3.9 × 10^–4^] followed by pseudobulbar affect [9/46 vs 71/776; 19.6% vs 9.1%; OR 2.4 (95% CI 1.1–5.2); *P* = 0.021] and face reflex [27/46 vs 322/776; 58.7% vs 41.5%; OR 2.0 (95% CI 1.1–3.7); *P* = 0.022], while no difference could be observed for palmomental sign. Regarding the burden of LMN signs, C9Pos and C9Neg patients did not show different MRC (Supplementary Fig. 1d) and LMNS scores (Supplementary Fig. 1e), except for a significantly higher frequency of tongue fasciculations and atrophy in the former group [28/46 vs 341/775; 60.9% vs 44.0%; OR 2.0 (95% CI 1.1–3.6); *P* = 0.025]. When considering HRE size, we did not observe any significant association with PUMNS, LMNS or MRC scores.

**Table 2:** Mean, median, standard deviation of PUMNS, LMNS, MRC values respectively in C9Pos and C9Neg patients

	C9Pos			C9Neg		SD	*P* value^a^
	Mean	Median	SD	Mean	Median		
PUMNS	129	12.5	7.6	10.0	9.0	7.3	0.010
Bulbar	1.9	2.0	1.3	1.4	1.0	1.2	0.005
Upper limbs	5.4	4.5	3.7	4.2	4.0	3.8	0.027
Lower limbs	5.7	6.0	3.6	4.5	4.0	3.6	0.023
LMNS	5.2	4.0	4.0	5.2	5.0	3.4	ns
Upper limbs	2.0	2.0	2.0	2.5	2.0	1.9	ns
Lower limbs	2.4	2.0	2.2	2.0	2.0	1.9	ns
MRC	49.0	52.0	10.0	49.1	52.0	10.3	ns
Upper limbs	25.2	26.0	5.3	24.0	26.0	6.2	ns
Lower limbs	23.8	26.0	6.5	25.2	28.0	6.3	ns

ns, not significant; C9Pos, patients carrying *C9orf72* repeat expansion; C9Neg, patients without *C9orf72* repeat expansion; PUMNS, Penn Upper Motor Neuron Score; LMNS, Lower Motor Neuron Score; MRC, Medical Research Council; SD, standard deviation

^a^Comparison between C9Pos and C9Neg patients

### Assessment of cognitive and behavioural phenotype

In our cohort, *C9orf72* HRE was significantly associated to comorbid behavioural variant FTD (bvFTD) [8/55 vs 22/905; 14.5% vs 2.4%; OR 6.8 (95% CI 2.9–16.2); *P* = 5.3 × 10^–7^] as determined by the Neary and Rascovsky criteria. ECAS was performed in a subset of 211 cases (22%) including 20 C9pos and 191 C9Neg patients, which were subsequently subdivided, according to the Strong revised criteria, in 85 unimpaired (40.3%), 41 ALSbi (19.4%), 52 ALSci (24.7%), 27 ALScbi (12.8%) and 6 ALSFTD (2.8%) patients. We did not observe a different distribution of HRE among these categories, apart from the ALSFTD group where C9Pos were significantly overrepresented [3/20 vs 3/191; 15.0% vs 1.6%; OR 11.1 (95% CI 2.1–59.1); *P* = 0.005]. Similarly, we did not find any difference in ECAS total, ALS-specific, ALS-nonspecific, and individual subdomain scores, either considering the raw scores or the normality cut-offs for the Italian population (Table 3).

**Table 3:** Cognitive and behavioral features of C9Pos and C9Neg patients

	C9Pos				C9Neg				*P* value^a^
	*n*	%	Mean	Median	*n*	%	Mean	Median	
executive			34.0	35.5			33.9	36.0	ns
fluency			17.1	20.0			16.1	18.0	ns
language			23.5	24.4			23.3	24.0	ns
ALS-specific			74.6	79.0			73.4	78.0	ns
memory			16.5	17.5			15.0	16.0	ns
visuospatial			11.1	11.0			11.4	12.0	ns
ALS-nonspecific			27.6	29.0			26.4	27.0	ns
ECAS total score			102.1	107.0			100.1	104.0	ns
dishinibition	5	29.4			8	4.7			1.6 × 10^–4^
apathy/inertia	5	29.4			167	37.7			ns
loss of sympathy/empathy	4	23.5			23	13.8			ns
perseverative/stereotyped/compulsive/ritualistic behavior	0	0.0			3	1.2			ns
hyperorality/altered food preferences	0	0.0			4	2.4			ns
ECAS behavioral symptoms			0.8	1.0			0.6	0.0	ns
FBI-A			2.5	2.0			2.3	1.0	ns
FBI-B			2.2	2.0			1.0	0.0	0.009
FBI			4.7	5.0			3.4	2.0	ns
BDI-II cognitive-affective			3.7	4.0			6.6	5.0	0.018
BDI-II somatic			5.5	5.0			7.6	7.0	0.042
BDI-II			9.2	7.0			14.2	13.0	0.015
STAI 1			45.2	42.0			50.8	49.0	0.008
STAI 2			45.1	43.0			50.2	49.0	0,033

ns, not significant; C9Pos, patients carrying *C9orf72* repeat expansion; C9Neg, patients without *C9orf72* repeat expansion; ECAS, Edinburgh Cognitive and Behavioural ALS Screen; FBI, Frontal Behavioural Inventory; BDI-II, Beck Depression Inventory-II; STAI, State-Trait Anxiety Inventory

^a^Comparison between C9Pos and C9Neg patients

**Fig. 3:**
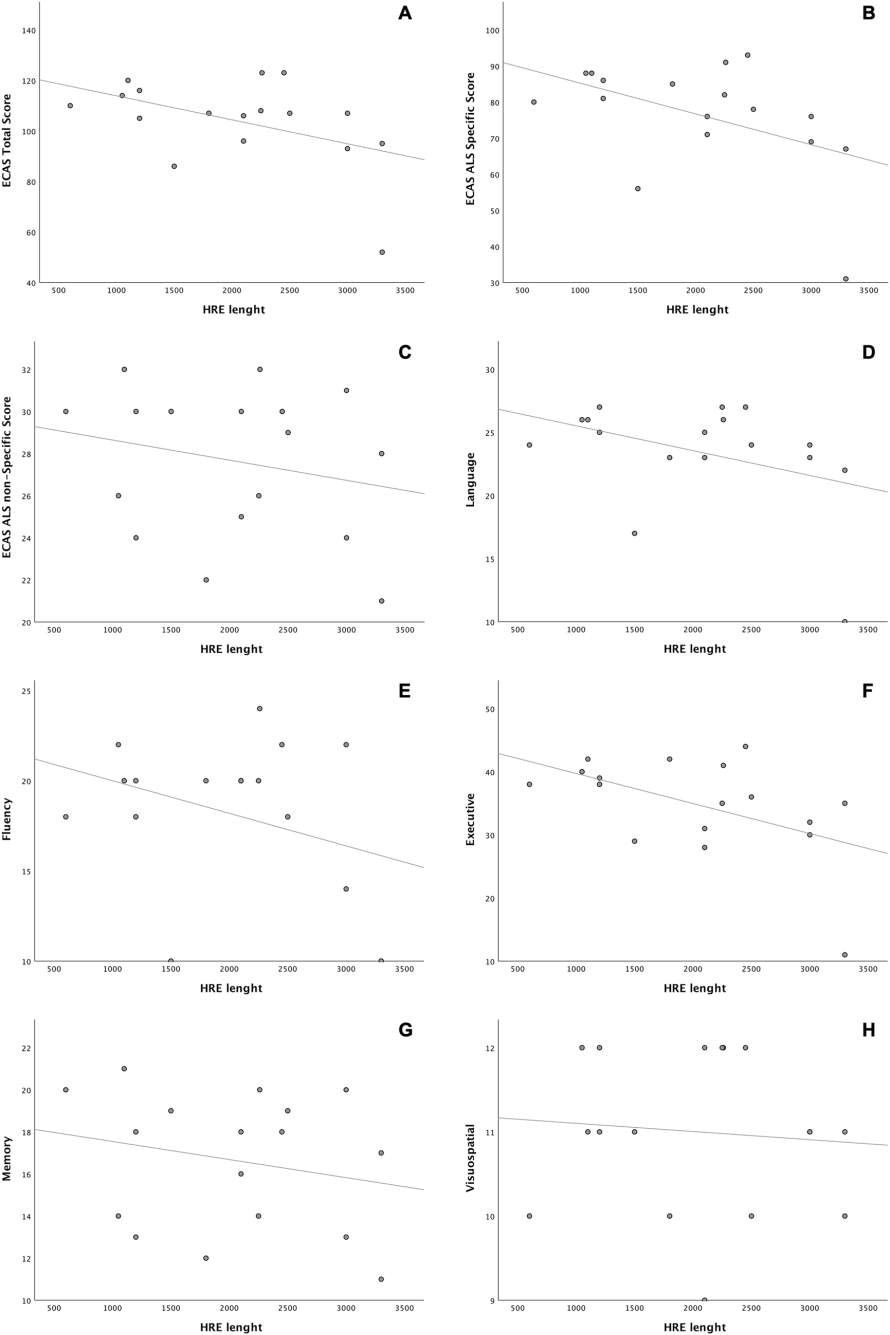
Correlation between hexanucleotide repeat expansion size and cognitive features. Simple dispersion with adjustment curve of hexanucleotide repeat expansion length, respectively for Edinburgh Cognitive and Behavioural ALS Screen (ECAS) total (*R*^2^ = 0.261, *P* = 0.036) (**A**), ECAS ALS-specific (*R*^2^ = 0.275, *P* = 0.031) (**B**), ECAS ALS-nonspecific (*R*^2^ = 0.048, *P* = NS) (**C**), ECAS language (*R*^2^ = 0.202, *P* = NS) (**D**), ECAS fluency (*R*^2^ = 0.125, *P* = NS) (**E**), ECAS executive (*R*^2^ = 0.320, *P* = 0.018) (**F**), ECAS memory (*R*^2^ = 0.035, *P* = NS) (**G**), ECAS visuospatial (*R*^2^ = 0.029, *P* = NS) (**H**) scores. Each dot represents an ALS patient. *HRE* hexanucleotide repeat expansion, *ECAS* Edinburgh Cognitive and Behavioural ALS Screen

Since Southern blot data were available for 17/20 C9Pos patients that underwent ECAS, the possible association between HRE size and cognitive features was tested. Interestingly, we found a modest, but significant inverse correlation between *C9orf72* HRE length and the ECAS total score (*R*^2^ = 0.261, *P* = 0.036). This phenomenon appeared to be mostly driven by worse performances at the ALS-specific tasks (*R*^2^ = 0.275, *P* = 0.031) and specifically at the executive subdomain (*R*^2^ = 0.320, *P* = 0.018). Within this subdomain, dysfunction at sentence completion (*R*^2^ = 0.404, *P* = 0.006) and social cognition (*R*^2^ = 0.250, *P* = 0.041) tasks seemed to account for the greater part of the impairment. These associations were still significant when accounting for age at DNA collection as a covariate. In contrast, no association was found between *C9orf72* HRE length and ALS-nonspecific cognitive domains (Fig. 3). We obtained a complete ECAS Carer Interview for 184/211 (87.2%) patients that underwent ECAS, including 17 C9Pos and 167 C9Neg cases. In this subgroup, the most common behavioural symptom was apathy/inertia (68/184, 37.0%), followed by loss of sympathy/empathy (27/184, 14.7%), behavioural disinhibition (13/184, 7.1%), hyperorality/altered food preference (4/184, 2.2%) and perseverative, stereotyped, compulsive or ritualistic behaviour (3/184, 1.6%). We did not find any difference between C9pos and C9Neg individuals in the total number of behavioural symptoms, nor with apathy, loss of sympathy, perseverative behaviour or hyperorality. Conversely, behavioural disinhibition occurred significantly more frequently in C9Pos patients [5/17 vs 8/167; 29.4% vs 4.8%; OR 8.2 (95% CI = 2.3–29.3; *P* = 1.6 × 10^–4^)], with two sub-items also showing a significant association with HRE [socially inappropriate behaviour: 2/17 vs 2/167; 11.8% vs 1.2%; OR 11.0 (95% CI 1.4–83.8); *P* = 0.004; impulsive, rash or careless actions: 3/17 vs 2/167; 17.6% vs 1.2%; OR 17.7 (95% CI 2.7–114.8); *P* = 7.1 × 10^–5^) (Table 3). These observations were further confirmed by the finding of higher scores at the FBI-B subscale, which explores the presence of positive symptoms such as behavioural disinhibition, among C9Pos individuals compared to C9Neg ones (2.2 ± 2.4 vs 1.0 ± 1.5, *P* = 0.009). Within this subscale, the items associated with HRE were judgment/impulsivity [3/13 vs 7/152; 23.1% vs 4.6%; OR 6.2 (95% CI 1.4–27.8; *P* = 0.033)] and inappropriateness [2/13 vs 2/152; 15.4% vs 1.3%; OR 13.6 (95% CI 1.7–106.3; *P* = 0.032)]. Conversely, we did not appreciate any association with the FBI-A, assessing negative symptoms (2.5 ± 2.0 vs 2.3 ± 2.8, *P* > 0.05), or with the FBI total scale (4.7 ± 3.9 vs 3.4 ± 3.8, *P* > 0.05) (Table 3, Supplementary Fig. 2a–c). The presence of psychotic symptoms was also similar in both groups (data not shown). We also analysed the presence and the severity of depression and anxiety symptoms using the BDI-II and STAI questionnaires. Interestingly, HRE was significantly associated with lower scores at STAI-Y1 (45.2 ± 6.9 vs 50.8 ± 9.9, *P* = 0.008), STAI-Y2 (45.1 ± 8.4 vs 50.2 ± 10.3, *P* = 0.033), BDI-II cognitive-affective (3.7 ± 3.1 vs 6.6 ± 5.8, *P* = 0.018), BDI-II somatic (5.5 ± 3.1 vs 7.6 ± 4.4, *P* = 0.042), and BDI-II total scores (9.2 ± 5.6 vs 14.2 ± 9.0, *P* = 0.015), indicating lower levels of self-reported depression and anxiety in C9Pos patients (Table 3, Supplementary Fig. 2d–h). However, no significant association was present between HRE size and the manifestation of behavioural, depression and anxiety symptoms.

## Discussion

In our single-centre analysis of 960 Italian ALS patients, we compared motor, cognitive and behavioural profiles between C9Pos and C9Neg individuals to define if these two groups have different phenotypes. Our results largely confirmed previous evidence from the literature. Indeed, our C9Pos patients showed a younger onset, higher odds of bulbar onset and a reduced survival [2, 4–8]. As expected, they also showed a higher prevalence of family history of ALS and dementia. On the contrary, no family history was observed for parkinsonian syndromes confirming the hypothesis that, although *C9orf72* HRE can be observed in ALS-plus phenotypes, the mutation is strictly related to TDP-43 proteinopathies [27, 28]. In our analysis, C9Pos patients presented with a more severe UMN involvement in all body regions and specifically in the bulbar segment. While different studies have reported the association between *C9orf72* HRE and bulbar onset [4–6, 8], ours is the first study to show a major burden of UMN involvement at the global and regional level in C9Pos patients.

As for the cognitive phenotype, C9Pos patients more frequently presented a phenotype within the FTD spectrum disorders as already described [2, 5, 8, 9]. The analysis of individual ECAS domains, however, did not show any qualitative difference between mutated and non-mutated patients, a result apparently in contrast with previous studies indicating a more severe impairment in executive functions and verbal memory in the former group [29]. However, when analysing the subgroup of patients for which Southern Blot was available, we found a statistically significant inverse correlation between HRE length and performance at the ECAS. This correlation was specifically relevant for ALS-specific cognitive functions, particularly for the executive function subdomain (sentence completion and social cognition tasks), thus suggesting a specific impairment in inhibitory cognitive components. Indeed, C9Pos patients with larger expansions had a more severe cognitive impairment, thereby supporting the emerging hypothesis that *C9orf72* HRE size may be a modifier factor of phenotype along the ALS-FTD clinical spectrum [30]. Given the difficulty in obtaining informative Southern blots for *C9orf72* HRE size and the high tissue mosaicism of the pathological expansion, conclusive evidence on the effect of HRE length on clinical phenotype, age of onset or disease duration is still lacking. Some studies describe significantly larger pathogenic HRE size in ALS patients compared to FTD cases [31, 32], which was not confirmed by other reports [33]. We too did not find any association with HRE length and disease manifestation in an Italian C9Pos pedigree presenting high intrafamilial variability [10]. In contrast to other repeat expansion disorders, the phenomenon of anticipation is still disputable since both expansions and contractions of the G_4_C_2_ repeat number have been described transgenerationally in C9Pos families [11, 34–36]. Also, with regard to disease duration, data are contrasting: some reports did not find any correlation with HRE size [31], while others observed that a longer expansion size was associated with shorter disease duration [32].

To our knowledge, very few reports have analyzed the differences in behavioral symptoms, degree of depression and anxiety between C9Pos and C9Neg patients. One study found that behavior was similarly impaired in both cohorts [29], while another report found increased apathy levels in C9Pos compared to C9Neg individuals [9]. Conversely, we did not appreciate any significant difference in the levels of apathy and other negative behavioural symptoms in our cohort. Remarkably, however, we found that behavioural disinhibition was significantly more present in C9Pos patients. This finding is further supported by the direct association between a more significant UMN impairment and the presence of behavioural symptoms [37], similarly to what observed in our cohort of C9Pos patients when compared to C9Neg ones. A previous study reported a higher presence of anxiety and depression in C9Neg vs C9Pos patients, even if the difference was not significant [29]. In a similar way, in our cohort C9Pos individuals showed significantly lower degrees of depression and anxiety when compared to C9Neg cases. This result could be interpreted as a loss of insight and a higher level of anosognosia in these patients. Loss of insight still represents an important under-recognized issue that should be measured within ALS patients’ current assessment [38]. Future studies on awareness and emotional processing in *C9orf72* patients will help to better clarify if the emotional response in these subjects is affected.

A limitation of our study is that ECAS was systematically performed in a sub-population of the whole cohort after the validation of the Italian version [19]. Despite this, no differences in demographic, clinical and motor features were observed between patients evaluated prior or after this timepoint (data not shown), suggesting that any bias due to missing data is limited. In fact, our study reports a consistent clinical description of a large cohort of Italian ALS patients in which C9Pos individuals were extensively characterized regarding the motor phenotype with the use of semiquantitative scales to assess the burden of UMN and LMN impairment. We also show for the first time that *C9orf72* HRE size may represent a modifier of cognitive phenotype along the ALS-FTD spectrum. It must be noted that our observations are based on blood DNA samples and given the occurrence of HRE mosaicism across tissues, they might be not fully representative of the HRE in affected cerebral tissues. Other possible limitations of our study are the absence of longitudinal data, the lack of correlation with neuroimaging markers and the analysis of a centre-based cohort rather than a population registry.

In summary, our results showed a distinct motor, cognitive and behavioural profile in ALS patients carrying the *C9orf72* HRE. We observed a significant association with the burden of UMN signs, the severity of ALS-specific cognitive impairment, as well as with the presence of positive behavioural alterations, such as disinhibition. Finally, anxiety and depression were uncommon in our C9Pos patients, and it is likely that the lower frequency of such features is related to reduced insight. A detailed motor, cognitive and behavioural assessment, including awareness and insight features, should thus represent a fundamental aspect of the clinical evaluation of ALS patients, specifically those carrying *C9orf72* HRE.

## Supplementary Information

Below is the link to the electronic supplementary material.

Supplementary file1 (DOCX 168 KB)
