# Correction to: Motor, cognitive and behavioural profiles of *C9orf72* expansion-related amyotrophic lateral sclerosis

**DOI:** 10.1007/s00415-023-11651-z

**Published:** 2023-03-13

**Authors:** Eleonora Colombo, Barbara Poletti, Alessio Maranzano, Silvia Peverelli, Federica Solca, Claudia Colombrita, Silvia Torre, Cinzia Tiloca, Federico Verde, Ruggero Bonetti, Laura Carelli, Claudia Morelli, Antonia Ratti, Vincenzo Silani, Nicola Ticozzi

**Affiliations:** 1 Department of Neurology and Laboratory of Neuroscience, IRCCS Istituto Auxologico Italiano, Università degli Studi di Milano, P.le Brescia 20, 20149 Milan, Italy; 2 Neurology Residency Program, Università degli Studi di Milano, Via Festa del Perdono 7, 20122 Milan, Italy; 3 “Dino Ferrari Center”, Department of Pathophysiology and Transplantation, Università degli Studi di Milano, 20122 Milan, Italy; 4 Department of Medical Biotechnology and Translational Medicine, Università degli Studi di Milano, 20122 Milan, Italy

**Correction to: Journal of Neurology (2023) 270:898–908** https://doi.org/10.1007/s00415-022-11433-z

The original version of this article unfortunately contained a mistake. There are corrections in the affiliations of the authors.

Original Affiliations.

Eleonora Colombo^1^ · Barbara Poletti^1^ · Alessio Maranzano^1,2^ · Silvia Peverelli^1^ · Federica Solca^1^ ·

Claudia Colombrita^1^ · Silvia Torre^1^ · Cinzia Tiloca^1^ · Federico Verde^1,2^ · Ruggero Bonetti^1,2^ · Laura Carelli^1^ · Claudia Morelli^1^ · Antonia Ratti^1,3^ · Vincenzo Silani^1,2^ · Nicola Ticozzi^1,2^

^1^ Department of Neurology and Laboratory of Neuroscience, IRCCS Istituto Auxologico Italiano, P.le Brescia 20, 20149 Milan, Italy.

^2^ “Dino Ferrari Center”, Department of Pathophysiology and Transplantation, Università degli Studi di Milano, 20122 Milan, Italy.

^3^ Department of Medical Biotechnology and Translational Medicine, Università degli Studi di Milano, 20122 Milan, Italy.

Correct Affiliations.

Eleonora Colombo^1^ · Barbara Poletti^1^ · Alessio Maranzano^2^ · Silvia Peverelli^1^ · Federica Solca^1^ ·

Claudia Colombrita^1^ · Silvia Torre^1^ · Cinzia Tiloca^1^ · Federico Verde^1,3^ · Ruggero Bonetti^2^ · Laura Carelli^1^ · Claudia Morelli^1^ · Antonia Ratti^1,4^ · Vincenzo Silani^1,3^ · Nicola Ticozzi^1,3^

^1^ Department of Neurology and Laboratory of Neuroscience, IRCCS Istituto Auxologico Italiano, P.le Brescia 20, 20149 Milan, Italy.

^2^ Neurology Residency Program, Università degli Studi di Milano, Via Festa del Perdono 7, 20122 Milan, Italy.

^3^ “Dino Ferrari Center”, Department of Pathophysiology and Transplantation, Università degli Studi di Milano, 20122 Milan, Italy.

^4^ Department of Medical Biotechnology and Translational Medicine, Università degli Studi di Milano, 20122 Milan, Italy.

