# Supplementary material for: Motor, cognitive and behavioural profiles of C9orf72 expansion-related amyotrophic lateral sclerosis
Source: J Neurol. 2022 Oct 29;270(2):898–908. doi: 10.1007/s00415-022-11433-z (PMC9886586; doi:10.1007/s00415-022-11433-z)
Supplement: Supplementary file 1 — Supplementary file1 (DOCX 168 KB) [file 415_2022_11433_MOESM1_ESM.docx]

**Supplementary Figure 1. Characterization of motor phenotype in C9Pos and C9Neg patients.**Distribution of total (*P* = 0.010) (**A**), bulbar (*P* = 0.005) (**B**) and spinal (*P* = 0.016) (**C**) PUMNS values, MRC score (*P* = NS) (**D**) and LMNS value (*P* = NS) (**E**) in C9Pos and C9Neg patients. For each group, the bold line shows the median, the grey box includes the middle 50% of the data and the whiskers show the minimum and the maximum values. Empty circle represents outliers. Kruskal-Wallis for independent samples.
Abbreviations: C9Pos = patients carrying C9orf72 repeat expansion; C9Neg= patients without C9orf72 repeat expansion; PUMNS = Penn Upper Motor Neuron Score; MRC = Medical Research Council; LMNS = Lower Motor Neuron Score.


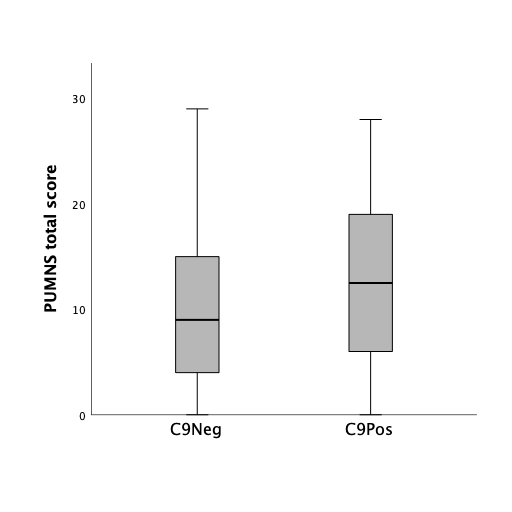

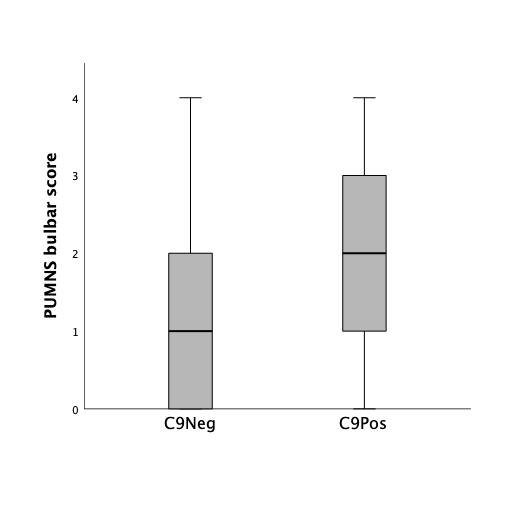

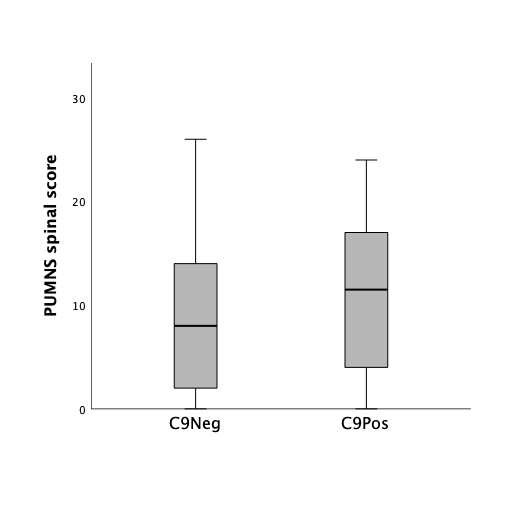

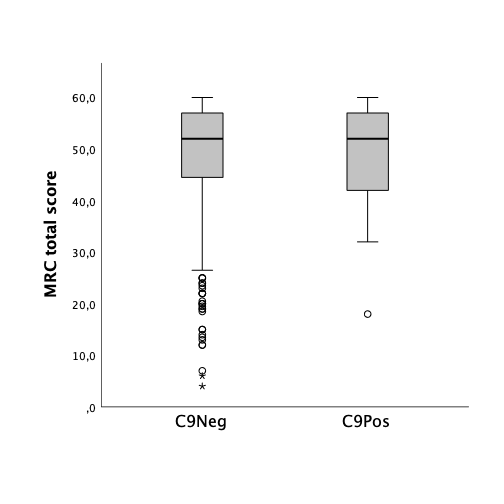

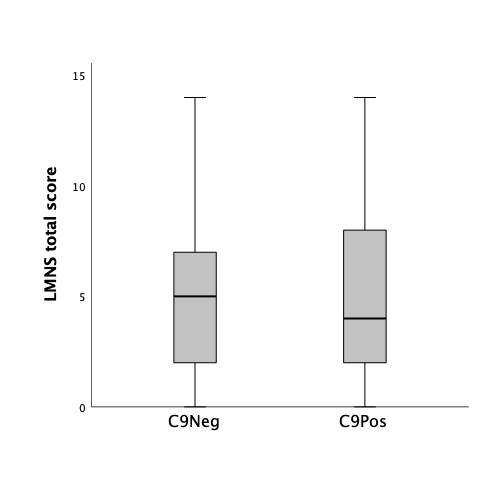


**E**

**D**

**C**

**B**

**A**

**Supplementary Figure 2. Characterization of behavioral phenotype in C9Pos and C9Neg patients.** Distribution of FBI part A (*P* = NS) (**A**), FBI part B (*P* = 0.009) (**B**), FBI total (*P* = NS) (**C**), BDI-II cognitive affective (*P* = 0.018) (**D**), BDI-II somatic (*P* = 0.042) (**E**), BDI-II total (*P* = 0.015) (**F**), STAI-Y1 (*P* = 0.008) (**G**) and STAI-Y2 (*P* = 0.033) (**H**) scores in C9Pos and C9Neg patients. For each group, the bold line shows the median, the grey box includes the middle 50% of the data and the whiskers show the minimum and the maximum values. Empty circle represents outliers. Kruskal-Wallis for independent samples.
Abbreviations: FBI= Frontal Behavioural Inventory; BDI-II= Beck Depression Inventory-II; STAI= State-Trait Anxiety Inventory; C9Pos=patients carrying C9orf72 repeat expansion; C9Neg= patients without C9orf72 repeat expansion.


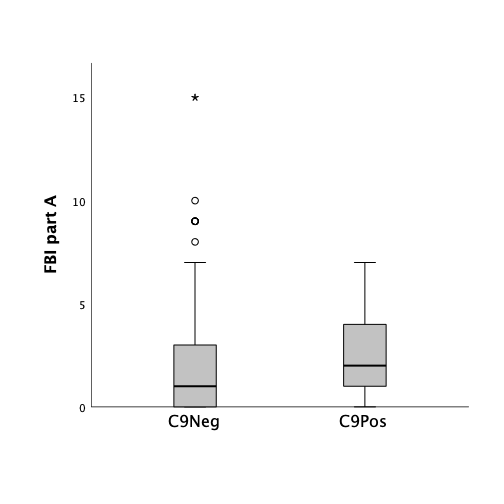

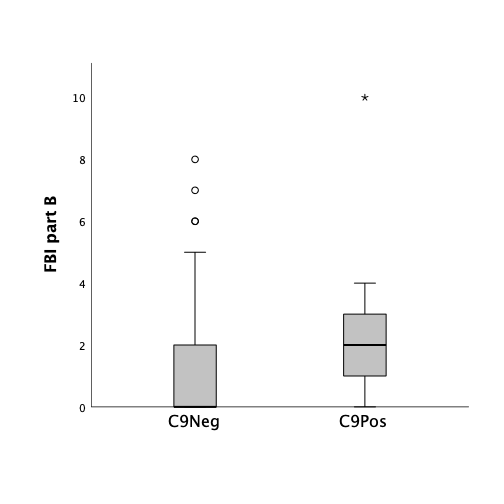

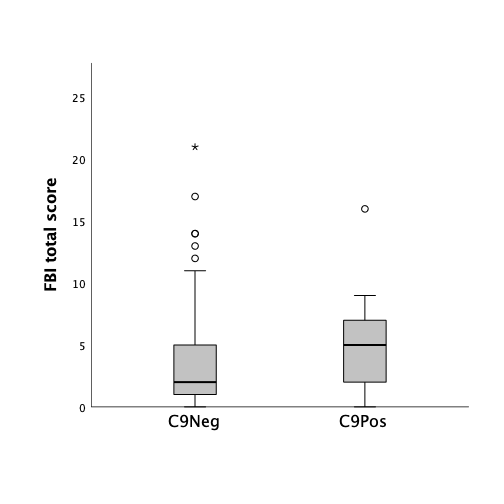


**C**

**B**

**A**

**
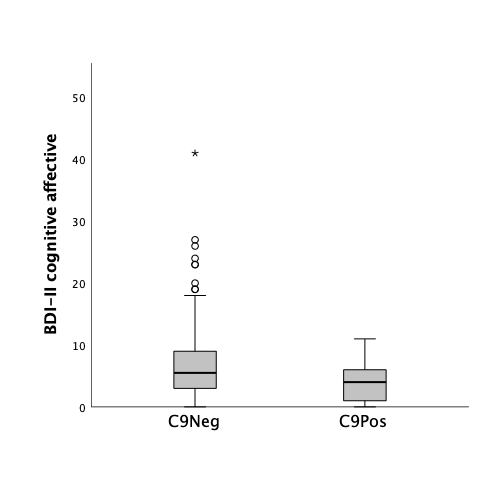
** **
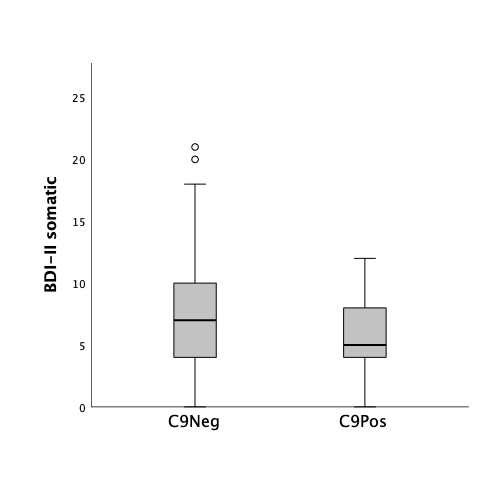
**
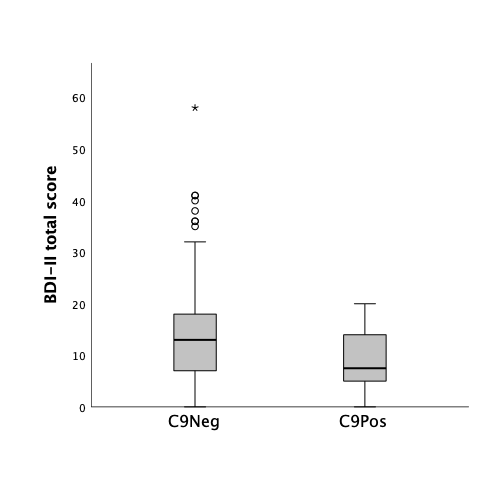


**F**

**E**

**D**


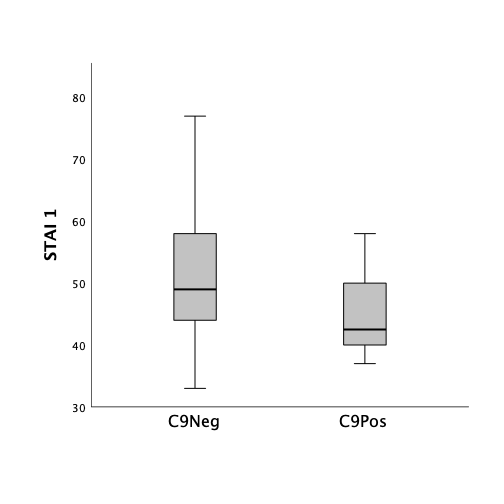

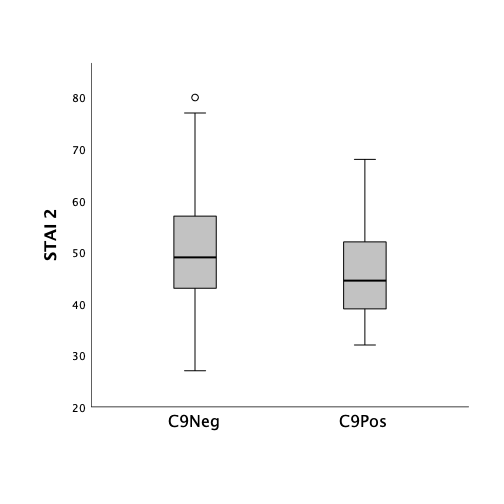


**H**

**G**

**Supplementary Table 1. Multivariate survival analysis using site of onset, age at onset and c9orf72 status as covariates. Female sex, spinal onset and C9Neg status were used as references**.
Abbreviations: ns=not significant; HR=hazard ratio; CI= confidence interval

| **Covariate** | **HR** | **95% CI inf** | **95% CI sup** | **p** |  | **reference** |
| --- | --- | --- | --- | --- | --- | --- |
| **Site of onset** | 1.19 | 0.96 | 1.48 | ns |  | spinal |
| **Age at onset** | 1.04 | 1.03 | 1.05 | 2.7x10^-23^ |  |  |
| **C9orf72 status** | 2.32 | 1.64 | 3.28 | 2.0x10^-6^ |  | C9Neg |
